# Comparative Aspects of Immunoglobulin Gene Rearrangement Arrays in Different Species

**DOI:** 10.3389/fimmu.2022.823145

**Published:** 2022-02-11

**Authors:** Marek Sinkora, Katerina Stepanova, John E. Butler, Marek Sinkora, Simon Sinkora, Jana Sinkorova

**Affiliations:** ^1^ Laboratory of Gnotobiology, Institute of Microbiology of the Czech Academy of Sciences, Novy Hradek, Czechia; ^2^ Department of Microbiology, University of Iowa, Iowa City, IA, United States

**Keywords:** B cell development, B cell receptors, cell differentiation, immunoglobulin heavy and light chains, gene rearrangement

## Abstract

Studies in humans and mice indicate the critical role of the surrogate light chain in the selection of the productive immunoglobulin repertoire during B cell development. However, subsequent studies using mutant mice have also demonstrated that alternative pathways are allowed. Our recent investigation has shown that some species, such as pig, physiologically use preferential rearrangement of authentic light chains, and become independent of surrogate light chains. Here we summarize the findings from swine and compare them with results in other species. In both groups, allelic and isotypic exclusions remain intact, so the different processes do not alter the paradigm of B-cell monospecificity. Both groups also retained some other essential processes, such as segregated and sequential rearrangement of heavy and light chain loci, preferential rearrangement of light chain kappa before lambda, and functional κ-deleting element recombination. On the other hand, the respective order of heavy and light chains rearrangement may vary, and rearrangement of the light chain kappa and lambda on different chromosomes may occur independently. Studies have also confirmed that the surrogate light chain is not required for the selection of the productive repertoire of heavy chains and can be substituted by authentic light chains. These findings are important for understanding evolutional approaches, redundancy and efficiency of B-cell generation, dependencies on other regulatory factors, and strategies for constructing therapeutic antibodies in unrelated species. The results may also be important for explaining interspecies differences in the proportional use of light chains and for the understanding of divergences in rearrangement processes. Therefore, the division into two groups may not be definitive and there may be more groups of intermediate species.

## Introduction

Immunoglobulin (Ig) gene rearrangement has evolved in all jawed vertebrates and involves recombination of variable (V), diversity (D), and joining (J) gene segments at their corresponding loci (reviewed in 1). The number of VDJ segments, their organization, orientation and position within the genome, and their frequencies utilized in B cells are known in many species. This is the result of modern genomic sequencing techniques and available and durable single-cell analyzes. Surprisingly, information on the mechanism by which they rearrange in these different species is sparse. In fact, they are based only on findings in mice and to some extent in humans (2), and it is assumed to be the same at least in mammals. The reason for this is understandable, because while genome sequencing is currently a straightforward task, uncovering the mechanism usually requires inbred animals in sufficient numbers and the technology of genetic modification. However, there are some exceptions such as our studies in swine where we characterized the development of B cells during ontogeny (3–6), their development in bone marrow (7), identified different developmental stages of B cells and the order and status of their IgH and IgL rearrangements (8), analyzed redundant rearrangements in the thymus (9), analyzed the order of IgLκ and IgLλ rearrangements during development (10), and showed the consequences of different rearrangement orders in recovered sequences (11) and IgH and IgL rearrangement configurations in individual peripheral B cells (12). These studies on non-transgenic and outbred animals were possible because of the organization of Ig loci and specific immunological properties. Pigs have a highly simplified IgH gene complex in which all V_H_ genes belong to the ancestral V_H_3 family sharing the same leader and framework sequences, and only one J_H_ segment is functional (8, 13, 14). Porcine IgL loci are also restricted to only two V_L_ families and only two functional J_L_ genes for both IgLκ and IgLλ (8, 15–18). Moreover, pigs possess an epitheliochorial placenta that prevents the prenatal transfer of maternal Ig (as well as smaller proteins) to the fetus (19, 20). This type of placentation, combined with prolonged gestation and numerous offspring, provides a favorable opportunity to characterize successive developmental steps during fetal life under naive conditions and without influence of extrinsic factors (4). In addition, pigs are precocial and do not require their mothers for survival. Late fetuses can be born aseptically into sterile isolators to easily produce germ-free piglets (18, 21, 22). Such germ-free animals are devoid of effector, memory, and plasma B cells, including long-lived bone marrow plasma cells that could interfere with developmental studies of naive B cells (5, 8, 12, 23, 24). Here we summarize our findings and compare them with results from other species to show that alternative pathways of V(D)J rearrangement are used.

## Review

### Mouse Paradigm of V(D)J Rearrangement

**Figure 1:**
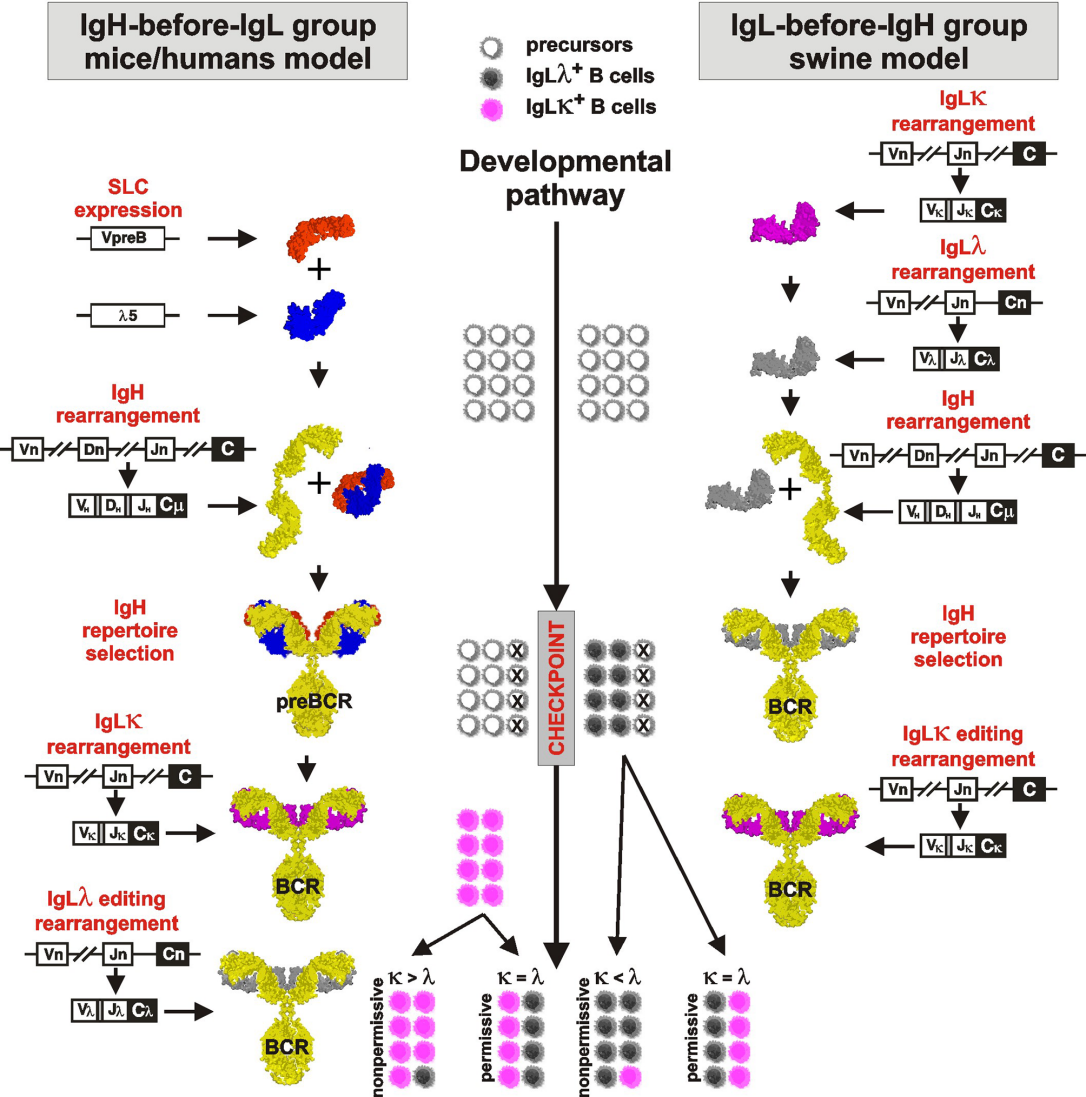
Proposed comparative model of B cell development in species rearranging IgH before IgL (left part) and IgL before IgH (right part). The critical checkpoint is indicated on the middle developmental line. Differently colored cells near the developmental line are for illustration only and indicate the proportional outcome of B cell development with respect to IgLλ^+^ (gray) and IgLκ^+^ (pink) B cells.

A model of B cell development and generation of B cell receptor (BCR) repertoire by V(D)J rearrangement is derived from mouse studies (reviewed in 1, 25). This sophisticated paradigm describes the rearrangement as a tightly sequential process regulated by a surrogate light chain (SLC) composed of λ5 (CD179b) and VpreB (CD179a). For overview of the process see **Figure 1**, left part. The first wave of the rearrangement occurs in the IgH locus of proB cells by the combinatorial joining of D_H_ to J_H_ segments on both chromosomes. The resulting preB-I cells subsequently rearrange a particular V_H_ segment to one incomplete DJ_H_ rearrangement on the first chromosome. Complete VDJ_H_ rearrangement for IgH is tested in preB-II cells for its productivity by the ability to form preBCR by association with pre-existing SLC (reviewed in 26). If IgH rearrangement is productive and can associate with invariant SLC, the resulting preBCR are anchored into the plasmatic membrane and associated with the signaling components CD79a and CD79b. This membrane complex delivers stop signals for further IgH rearrangement ensuring the selection for productive rearrangement and allelic exclusion (27). If IgH rearrangement is not productive and/or fails to fold correctly with SLC, the cell has one more chance to rearrange V_H_ to DJ_H_ using the second chromosome. The large preB-II cells die in case of failure but survive, expand, and consecutively become small preB-II cells in case of success. Up to this stage of development, no IgL rearrangement takes place. IgL locus is rearranged only if productive IgH was successfully tested by SLC and the IgH loci are closed for further rearrangement.

IgL rearrangement thereafter begins in surviving small preB-II cells initially with IgLκ, which continues to rearrange until it is productive and forms an authentic BCR (28). Multiple IgLκ rearrangements (editing) are possible because IgL genes, unlike the IgH locus, do not contain a D segment and do not lose recombination signal sequences between unused V_L_ and J_L_ segments. Rearrangement in the IgLκ loci is finished when (1) any IgLκ protein can form an authentic BCR with existing IgH and small preB-II cells become immature B cells or (2) all functional Vκ and/or Jκ segments on both chromosomes have been used and/or IgLκ loci have been inactivated. Inactivation of IgLκ occurs by excision of Cκ segments from the genome by recombination of any remaining Vκ segment or upstream Cκ recombining element (RE) to downstream recombining sequence in mice or κ deleting element in other species (hereafter referred to as KDE recombination) (28, 29; reviewed in 30). The existence of KDE has been demonstrated in all species studied, and the ablation of Cκ segments by Vκ-KDE and RE-KDE recombination before any IgLλ rearrangement is thought to ensure the isotypic exclusion (30).

### Swine Deviations From Mouse Paradigm

Porcine B cell development begins with IgL rearrangement in the absence of IgH rearrangement or components of SLC (VpreB and λ5) (8, 12). For overview of the process see **Figure 1**, right part. Similar to mice, IgLκ rearrangement is a beginning, but it occurs only on the first chromosome and the precursors become IgLκ^+^IgLλ^—^IgH^—^. There are no IgH rearrangements in these precursors yet, and if some occur rarely, if they are productive, and if they match productive IgL, the precursors become immature B cells expressing BCR. This is only a small fraction of the final IgLκ^+^ B cell pool as evidenced by cultivation and sorting studies (10). In the absence of IgH, the vast majority of the remaining cells rapidly replace the initial IgLκ rearrangement with successive IgLλ rearrangement. These precursors thus develop from IgLκ^+^IgLλ+IgH^—^ to IgLκ^—^IgLλ+IgH^—^ precursors, which continue to rearrange (and consume) further Vλ genes until IgH rearrangement occurs. As indicated by sorting and sequencing studies (11), most of the initial IgLκ genes are inactivated by KDE recombination. Rearrangement of IgH occurs at the next developmental stage and follows the same rules as known from mice: Incomplete DJ_H_ rearrangements are primarily formed on both chromosomes followed by complete VDJ_H_ on the first chromosome (7, 9). The IgH product is tested for its productivity and ability for surface expression by pairing with pre-existing authentic IgLλ. In case that both IgH and IgLλ rearrangements are productive, the cells become immature IgLλ^+^ B cells. The probability of productive IgLλ is very high because early rearrangements are direct VJ joins (31–33). The probability of productive IgH rearrangement follows the 1/3 success rule due to shifted reading frames (3, 7, 9). In the case of defective IgH, there is one more chance to rearrange V_H_ to DJ_H_ using the second chromosome. If successful, these early immature B cells survive and expand (8). Since initial IgLκ rearrangement was replaced by successive IgLλ rearrangement before IgH rearrangement, the early immature B cells are almost exclusively IgLλ^+^. This is substantially different from mice, in which IgLκ^+^ B cells are generated earlier (28). The generation of IgLκ^+^ B cells in porcine bone marrow occurs during the transition to late immature B cells by rearrangement of germline IgLκ genes on the second chromosome (10). During this process, which gives rise to the majority of immature IgLκ^+^ B cells, the existing IgLλ rearrangement is silenced but remains in the IgLκ^+^ B cells. This is another principal difference from mice because the vast majority of IgLκ^+^ B cells carry silenced and mostly productive IgLλ transcripts (10, 11). This peculiarity can be traced even in peripheral mature B cells (12). There may even be additional IgL editing in a small fraction of late immature cells if the second wave of IgLκ rearrangement is replaced by secondary IgLλ rearrangement. In this case, the existing secondary IgLκ rearrangement is again inactivated by KDE recombination to allow secondary IgLλ rearrangement (10–12).

### What Is Essential and What Optional

Studies in pigs have shown that some steps in the sequential process of V(D)J rearrangement are essential, whereas others are optional. The first essential process is that the IgH and IgL loci rearrange at different developmental stages. Thus, the process is sequential, but the respective order may vary (see below). This may be related to a reduction in the number of IgL isotypes during evolution, resulting in only one or two isotypes in birds and mammals. These can be more comfortably controlled than in skates and sharks, which have multiple IgH and IgL loci rearranged simultaneously (34, 35). In any case, once IgH rearrangement begins, it occurs consecutively on the first chromosome and, if not productive, also on the second chromosome, otherwise the developing B cell dies. Our analyses of single cells have never revealed two productive IgH rearrangements (9, 10). Furthermore, the developmental stage at which IgH rearrangements occur is also a crucial checkpoint of differentiation regulated by intrinsic factors of the bone marrow (7) always followed by several rounds of proliferation to increase a cohort of precursor B cells with identical IgH chain (reviewed in 36). Another essential step is also a preferential rearrangement of IgLκ before IgLλ. This has been demonstrated in many other species (37) and the positions/organizations of κ-enhancers are highly conserved (30). The function of KDE recombination is also critical. Inhibition of IgLκ by Cκ excision occurs before the switch to IgLλ rearrangement.

On the other hand, an optional process includes the independence of IgH and IgL gene rearrangements so that the respective order may vary. While IgH precedes IgL rearrangement in mice, IgL precedes IgH rearrangement in swine. Interestingly, the independence of IgH and IgL rearrangement was predicted based on results with virus-transformed preB cells (38) and demonstrated in IgH deficient mice, which rearrange IgL at normal frequencies and with normal kinetics (39, 40). It has also been demonstrated that a small fraction of developing B cells can rearrange IgL before IgH even in mice under physiological conditions (41). Swine can do this regularly under physiological conditions (8, 10). It should be emphasized that there may also be an intermediate group of animals that rearrange IgH and IgL competitively. An example of this is birds that do it very early in fetal life (42–44). Another optional process is the independence of IgLκ and IgLλ rearrangements on different chromosomes. Although IgLκ rearrange preferentially as explained above, and both IgLκ loci are consumed before any IgLλ rearrangement in mice, IgLκ and IgLλ rearrangements on different chromosomes may occur at different developmental stages in pigs. As also demonstrated in swine, IgL editing can occur much later in immature B cells, which is also true for any other species because BCR editing is used in the establishment of central tolerance in late immature B cells (36). The other optional process is the use of SLC to select productive IgH rearrangements. Pigs use an authentic IgL and do not need SLC.

Certainly, there are still some unresolved issues, such as whether incomplete DJ_H_ rearrangements on both chromosomes occur in the earliest precursors in all species. In swine, they do as in mice and humans (7, 8). We have also never observed multiple D_H_ rearrangements in our sequences. However, V_H_ to D_H_ rearrangement may precede D_H_ to J_H_ rearrangement in rabbits (45). In chickens, multiple D_H_ to DJ_H_ rearrangements have been reported before a subsequent rearrangement to the V_H_ gene (46).

It needs to be emphasized that it is not the intention of this report to discuss the molecular mechanisms of the V(D)J rearrangement machinery. It is apparent that essential components are also critical enzymes, such as the recombinase-activating genes (RAG), which are required for DNA cleavage, or the terminal deoxynucleotidyl transferase (TdT), which can facilitate N-nucleotide additions. As indicated by genome sequencing, this is also true for recombination signal sequences (RSS) and regulatory factors required for rearrangement processes. The encyclopedic information on these aspects can be found in other reviews.

### Controversial Function of SLC

Originally, SLC was expected to ensure the allelic exclusion. This was disproved by the construction of knockout mice in which different components of SLC were deleted but the exclusions remained intact (47, 48). Only targeted disruption of the membrane exon of IgH genes results in allelic inclusion (27, 49, 50), confirming that anchoring of IgH in the cell membrane is essential. Experiments with transgenic mice have also demonstrated that SLC is not even required for the selection of productive IgH. The deficiency in SLC does not prevent the initiation of IgL rearrangement or the development of mature B cells (47, 48). Although mice lacking SLC have somehow reduced number of peripheral B cells, they have normal serum IgM levels (47) and immune responses (48). All these experiments suggest that the SLC play only a role in increased efficiency of B cell generation and faster membrane deposition of successfully rearranged IgH in the absence of IgL. The SLC is not necessary if IgL is already present, which happens in all B cells with both IgH and IgL successfully rearranged. Since IgH and IgL rearrangements are independent (38–41), the SLC is not necessary if the order of IgH and IgL rearrangement is reversed.

There are three VpreB in mice (VpreB1, VpreB2, VpreB3) and two in humans (VpreB1 and VpreB3). While VpreB1 and VpreB2 are co-expressed and serve for IgH selection (1), the role of VpreB3 is different and probably interacts with IgH in the endoplasmatic reticulum (26, 51). However, recent studies in several species indicate that the VpreB and λ5 genes may function in other processes than the formation and testing of IgH. In chickens, no homologues of λ5 have been identified and the function of VpreB3 in these animals is the retention of free IgL inside of cells (51). Cows have all three VpreBs but VpreB2 and VpreB3 have biological functions unrelated to B cells development (52). Marsupials have only maintained VpreB3 and do not have VpreB1, VpreB2, or λ5 in the genome (53). Pigs have VpreB1, VpreB3, and λ5 in the genome (10) but these are not used for IgH selection (8, 10) and they are mainly expressed in non-lymphoid cells (8, 10, 33, 54). It is therefore possible that VpreB probes the fitness of other molecules as well, and that its usage in mice for the selection of productive IgH rearrangements is a highly specialized role adopted by only some species.

### Expression of IgL on the Cell Surface Without IgH

Initial IgL rearrangements in the absence of IgH can be expressed on the cell surface of early precursors in swine (8, 10). This is a striking observation, as it is generally assumed that IgL cannot anchor to the cell membrane without IgH. However, free IgL are common in human pathogenesis, and these so-called Bence Jones proteins have been known for >170 years (55). This demonstrates that IgL are able to escape from the endoplasmatic reticulum without being chaperoned by IgH. Free IgL in humans are mostly a product of plasma cells in which IgL are produced in excess to IgH (56). This is understandable because humans use IgH before IgL rearrangement and the IgL produced are likely to be in excess only in plasma cells. On the other hand, mice do not produce Bence Jones proteins under normal conditions, indicating a different kind of regulation for IgL synthesis. Early porcine precursors do not have IgH, so IgL is present in excess until IgH rearrangement occurs. Unfortunately, the mechanism by which free IgL attaches to the surface is not fully known. The vast majority of studies investigate the secreted free IgL. However, surface expression of free IgL has been demonstrated in virus-transformed preB cells, which also showed that free IgL do not associate with other proteins (38). More sophisticated studies showed that free IgL associates with the outer membrane *via* interaction with phospholipids such as sphingomyelin A (57). Importantly, free IgL are only associated with the surface of cells that produce these IgL (57). Our results also exclude the possibility that free IgL on a surface may be acquired incidentally from other sources (8).

### The Role of KDE, IgL Isotypic Exclusion and Distribution of IgL Rearrangements in B Cells

Preferential usage of IgLκ rearrangements on both chromosomes in mice (37) and the mechanism of IgLκ inhibition by KDE recombination prior to any IgLλ rearrangements (30) have four important consequences: (1) IgLκ^+^ B cells are generated earlier, (2) IgLκ^+^ B cells highly predominate over IgLλ^+^ B cells, (3) IgLκ^+^ B cells have both IgLλ loci in the germline, while (4) IgLλ^+^ B cells have rearranged IgLκ loci inactivated by Cκ ablation (28, 58). This is true and evident in mice, which have hundreds of Vκ genes and generate >90% of IgLκ^+^ B cells. Indeed, only a few Vκ and Jκ genes are required for productive IgLκ rearrangement because the 1/3 chance for out-of-frame rearrangement can be overcome by about three successive rearrangements and only on one chromosome. However, the proportional usage of IgLκ and IgLλ genes is not the same in all species, and some use >90% IgLλ (see below and **Table 1**), which is not easily explained by preferential IgLκ rearrangement and KDE recombination.

**Table 1:** Number of biologically functional (and total) gene segments in different species*.

Species	Vκ IGKV	Jκ IGKJ	Vλ IGLV	Jλ IGLJ	V_H_ IGHV	D_H_ IGHD	J_H_ IGHJ	IgLκ usage	SLC (VpreB/λ5)
Mice	**80** (>100)	**4** (5)	**2** (2)	**4** (4)	**>100** (>100)	**16** (31)	**3** (4)	95%	YES, B cell genesis
Rats	**>100** (>100)	**5** (6)	**8** (10)	**2** (3)	**>100** (>100)	**25** (35)	**4** (4)	90%	?
Humans	**44** (>100)	**5** (5)	**32** (>100)	**4** (6)	**45** (130)	**27** (30)	**6** (9)	60%	YES, B cell genesis
Pigs	**10** (14)	**2** (5)	**10** (23)	**2** (4)	**10** (25)	**2** (4)	**1** (5)	50%	YES, other processes
Goats	**6** (15)	**1** (4)	**25** (63)	**1** (2)	**4** (34)	**2** (4)	**1** (6)	20%	?
Horses	**19** (60)	**4** (5)	**27** (144)	**4** (6)	**4** (50)	**35** (40)	**8** (8)	7%	?
Sheep	**8** (13)	**1-NC?^#^** (3)	**14** (43)	**1** (2)	**6** (10)	**4** (2)	**2** (6)	5%	?
Cattle	**6** (25)	**1-NC^#^** (4)	**24** (63)	**5** (8)	**10** (36)	**9** (23)	**2** (4)	5%	YES, B genesis?, other processes
Marsupials	**37** (122)	**2** (2)	**35** (64)	**8** (8)	**21** (25)	**3?** (3)	**2** (6)	35%	NO, other processes
Chickens (birds)?	**0**	**0**	**1** ^$^ (200)	**1** (1)	**1** ^$^ (100)	**16** (16)	**1** (1)	0%	NO, other processes
Bats	**0**	**0**	**93** (>100)	**7** (80)	**66** (77)	**8** (8)	**7** (9)	0%	NO, other processes

Data are based on our results and other sources (25, 53, 59–64). The values in bold represent the number of biologically functional genes and the values in parenthesis represent the total number of genes.

*Numbers are approximate because genome assemblies may not be finished and biological functionality not fully proved. Functional genes do not correspond to IMGT because functionality in IMGT is based on sequences and mostly not tested for expression.

^#^NC means that putatively functional genes have noncanonical RSS so that rearrangement is highly inefficient or impossible.

^$^Chickens use gene conversion and pseudogenes can be used in functional rearrangements.

^?^Unknown or uncertain.

In any case, KDE recombination (leading to Cκ ablation and inhibition of IgLκ genes before IgLλ rearrangement) appears to be an effective mechanism to achieve isotypic exclusion. Such an exclusion function would be of particular interest given that KDE recombination is highly conserved in many (if not all) species (30). On the other hand, studies in humans showed the co-expression of multiple IgLκ and IgLλ rearrangements in different combinations in single B cells (65). Studies in mice showed that IgLκ production does not inhibit secondary rearrangements and multiple productive IgL can be detected in single cells (66). Studies in pigs extended these findings from the perspective of species which use IgL before IgH rearrangement and confirmed that multiple and even productive IgLλ rearrangements are present in IgLκ^+^ B cells (8, 10–12). Moreover, multiple IgL rearrangements can be effectively transcribed in a single cell (12, 65). Also, not all IgLκ^+^ B cells undergo Cκ deletion by KDE recombination on the first chromosome before rearranging on the second (10, 66). The coexistence of multiple productive IgL rearrangements in a single cell is highlighted in fish (67), where KDE cannot control up to four different IgL types encoded by distinct C_L_ genes (18, 34). Therefore, the function of KDE in isotypic exclusion is implausible. More probably, KDE recombination supports efficient switching from IgLκ to IgLλ rearrangement, but has no control over which IgL allele is rearranged, whether it is productive, transcribed, and expressed. Our sorting data suggest that unused IgL rearrangements are silenced in translation or in export of IgL protein because they have the corresponding mRNA but are not expressed on the surface (10). Silencing of the IgH locus is partially known (68) and is ensured by nonsense-mediated decay (NMD) (69). Whether a similar mechanism operates for IgL is unknown, namely because different IgL are located on different chromosomes and can be silenced even when they are productive. Some reports indicate that silencing operates after translation on a “best-fit, best-serve” basis when transcription and translation of unused IgL is at a very low level. This would be consistent with findings in mice (66).

It must be emphasized that the final KDE recombination depends on the available number of Jκ rather than Vκ genes, since the number of possible successive rearrangements is limited by the smaller number. If a given species has only one functional Jκ, the further editing is only KDE-mediated Cκ ablation. This is not at all uncommon as **Table 1** shows. Goats, sheep, and cattle have only one functional Jκ among several other nonfunctional ones (59). Furthermore, the remaining Jκ in cattle and sheep has a noncanonical RSS (59) that may favor immediate KDE-mediated ablation because initial functional rearrangement is inefficient or impossible. This could lead to the elimination of almost all IgLκ rearrangements and be only the final step before the evolutionary loss of all IgLκ usage, as occurred in bats (60, 61).

### Can Authentic IgL Be Used for Selection of Productive IgH Repertoire?

SLC is invariant and therefore always “productive”. It can theoretically bind to any productive IgH rearrangement to ensure its selection. This appears as a huge advantage over authentic IgL because the initial IgL rearrangements in precursors may be out of frame or nonproductive for other reasons, such as internal stop codons. However, investigation in mice has shown that as many as 50-70% of productive IgH fail to pair with SLC and developing cells become apoptotic (**48**; reviewed in **1**). On the other hand, experiments in pigs have shown that the initial IgL rearrangements used for selection of subsequent IgH rearrangements are >88% in-frame, have no mutations, and no N-additions (11). Such success rate for authentic IgL is considerably higher than has been reported experimentally for SLC. Authentic IgL use also different V_L_ and J_L_ genes and could allow the generation of B cells whose IgH would not be capable of pairing with always the same but always imperfect SLC (48). Therefore, the IgH repertoire selected by authentic IgL should not be biased by the existence of one type of non-polymorphic peptide chains like SLC, but selection is driven by a germline authentic IgL. In fact, each type of IgL with its specific sequence could serve as a different type of SLC, so selection driven by authentic IgL could have a substantial advantage over SLC, and apoptic turnover could be even lower. Repeated experiments have shown that in the absence of antigenic pressure (under fetal and germ-free conditions), both IgLκ and IgLλ rearrangements are direct IGLV-IGLJ joins without deletions and N additions (32, 33).

### Efficiency of B Cell Generation and Evolution of Ig Rearrangement

Based on current knowledge, it is difficult to estimate whether the use of authentic IgL is less redundant and more efficient than SLC-dependent selection (see section above). The established order of IgH before IgL gene rearrangement seems to be the most ancestral because this strategy is used in amphibians (70). However, amphibians and fish do not use SLC and the number of B cells per gram of body mass is > 10-fold lower than in birds or mammals (70, 71). A similar effect is seen in marsupials, which also keep IgH before IgL rearrangements and lack SLC (53). These mammals have minimal levels of serum antibodies even several months after birth (72), which is comparable to the kinetics of mice with genetically deficient SLC (48). Thus, one group of successor animals could increase the efficiency of B cell generation by employing the existing components of SLC. Others might reverse the order of IgH and IgL rearrangement and omit the SLC requirement or employ yet other mechanisms such as the gene conversion in chickens (44). The reason why some species do not retain the ancestral IgH before IgL rearrangement may be due to their limited IgH repertoire (62). Chickens have only a single functional V_H_ and J_H_ segment (44) and pigs are restricted to ten closely related V_H_ and one J_H_ segment (reviewed in 18, 22). Although it has been demonstrated that CDR3 junctional diversity can compensate for the limited combinatorial repertoire (13, 18), limited or highly homologous V_H_ genes can also be a limiting factor when invariant SLC does not associate with them (48). This may also be a reason why species with limited V_H_ repertoire have a much higher number of V_L_ segments in the genome (61).

The explanation for the different efficiency of B cell generation can be the critical checkpoint at a developmental stage where IgH rearrangements occur. This checkpoint has been characterized in mice (73; reviewed in 36), humans (37), and also in swine (7). In mice and humans, the checkpoint is overcome by expression of functional preBCR (74). The same checkpoint occurs in porcine IgL^+^ precursors during IgH rearrangement and is overcome by expression of authentic BCR (7, 8). In all species, the checkpoint is regulated by intrinsic factors of the bone marrow (or appropriate stromal cells), and it is followed by several rounds of proliferation to increase a cohort of precursor B cells with identical IgH chains. However, the generation of B cells without the bone marrow is still possible but very inefficient (3, 74), as in the case of the variants described in the above paragraph.

Two important observations were made in experiments with porcine bone marrow stromal cells: the first confirms that B cells developing in the absence of stromal cells contain the IgH rearrangement on only one chromosome, while stromal cells support the rearrangement on both chromosomes (7). This phenomenon was previously observed *in vivo* during early ontogeny, when B cells developing in the yolk sac and fetal liver prior to a functional bone marrow were rare and had only a single productive IgH rearrangement (3). Such an observation cannot be made in mice because maturation of B cells in the fetal liver of mice coincides with maturation in the bone marrow, while in fetal pigs there is a 25-day window in between (4). These results collectively indicate that the opening of the second chromosome for rearrangement does not occur in the absence of the bone marrow. The second observation confirms that the absence of stromal cells leads to the accumulation of IgLλ^+^IgH^—^ precursors and the preferential generation of IgLλ^+^ B cells (8, 10). This is also exactly what happens *in vivo* during early ontogeny, when IgLλ transcripts are about 20-times more frequent than IgLκ (54, 75). The apparent absence of IgLκ transcripts in the yolk sac and fetal liver led us formerly to the incorrect conclusion that IgLλ might precede the rearrangement of the IgLκ genes in pigs (54). Differences in the ability of the bone marrow to support B cell development throughout the checkpoint or its timing can therefore explain interspecies differences in the ration of IgLκ/IgLλ usage (see below).

### IgLκ to IgLλ Ratio in Different Species

As mentioned earlier, >95% of mouse B cells are IgLκ^+^. However, humans and pigs have about the same amount of IgLκ^+^ and IgLλ^+^, while species like cows, sheep, horses, dogs, and cats have >90% of IgLλ^+^ B cells (63). The enormous interspecies differences are often explained by the disproportionate number of Vκ and Vλ genes in a genome, which in some cases corresponds to the expressed IgLκ/IgLλ ratio (25, 64) but in others does not (61); see **Table 1**.

However, the preferential IgLκ rearrangements on both chromosomes and the mechanism of KDE recombination do not allow the generation of substantial numbers of IgLλ^+^ B cells (see above), especially in species that have relatively high numbers of functional Vκ genes such as horse (19), pig (10), sheep (8), cat (12) or dog (19) (**Table 1**; www.imgt.org). According to the results from swine, the difference in the use of IgLκ compared to IgLλ is more likely explained by the sequence of IgL rearrangements on different chromosomes and/or the permissiveness of the microenvironment to support efficient B cell development. Although IgL rearrangement begins with IgLκ and progresses to IgLλ probably in all mammals (28, 29), the outcome may be the result of two different processes (1): secondary IgLκ is not consumed before IgLλ rearrangement and/or (2) secondary IgLκ rearrangement is not permitted in given developmental step. In species that use IgL before IgH rearrangement such as swine, these possibilities lead to early genesis of IgLλ^+^ B cells, while most IgLκ^+^ B cells are generated later and require permissive bone marrow stromal cells (10). Therefore, the dozen-fold prevalence of early IgLλ^+^ B cells is compensated to approximately 1:1 ratio of IgLλ:IgLκ in immature B cells (10). If the bone marrow is less permissive, the ratio may be less favorable for IgLκ^+^ B cells, and if it is not permissive, the majority of all B cells would be IgLλ^+^. This could be the case in some IgLλ-high species, in which the lymphoid potential of the bone marrow declines with age (52). The same phenomenon can be observed artificially in transgenic mice that have a longer time for successive IgL rearrangement, resulting in a considerable shift for higher usage of IgLλ over IgLκ (76). In species that use IgH before IgL rearrangement, such as humans, the same possibilities may lead to the protracted or suspended second wave of IgLκ rearrangement or accelerated IgLλ rearrangement. This can be demonstrated by analyses of IgLκ rearrangements in IgLλ^+^ B cells. Mouse IgLλ^+^ B cells are generated as a last chance for developing precursors when IgLκ loci are exhausted (66). On the other hand, a substantial number of human IgLλ^+^ B cells may have IgLκ loci in the germline or rearranged, and only about half of them have IgLκ genes deleted (28, 73). The effect of rearrangement order and the permissivity to support efficient B cell development is indicated in middle part of **Figure 1**.

Additional evidence that the timing and sequence of each rearrangement control the proportional utilization of IgL genes comes from the proportional usage of the different V_L_ and J_L_ segments in different species. The majority of mouse IgLκ^+^ B cells preferentially use 3’ Vκ and 5’ Jκ genes, while more 5’ Vκ and 3’ Jκ segments are used in IgLλ^+^ B cells (77, 78) since a pre-existing VJ_L_ can only be edited by a rearrangement that uses more 5’ V_L_ along with more 3’ J_L_ genes. However, when SLC is inactivated in transgenic mice, they use more 5’ Vκ genes (78) and resemble more swine, which also do not use SLC. The situation in mice is also different from humans, in which both naive and experienced B cells preferentially use more 3’-Vκ genes (79). However, more 5’ Vλ genes are used than even in the naive human repertoire (80), likely reflecting a more independent IgLλ rearrangement (28, 73). In any case, the situation is quite different in the pigs, which have been repeatedly reported to use more 5’ V_L_ genes in both IgLκ (16) and IgLλ (15, 32, 33, 81). This discrepancy is mainly caused by the early use of IgLκ rearrangement in precursor cells, which is replaced by IgLλ rearrangement before IgH rearrangement (11). The depletion and inhibition of porcine Cκ genes on the first chromosome during this early development has another fundamental limitation: only IgLκ genes on the second chromosome can be used to generate IgLκ^+^ B cells; and pigs have only two functional Jκ segments on one allele (18). Such restriction to only two consecutive IgLκ rearrangements is probably the reason why only two major Vκ genes are preferentially used in swine (11, 32) and why the Vκ repertoire is severely restricted compared to Vλ (32, 33). Higher flexibility and diversity of IgLλ than of IgLκ has also been found in other λ-high species (61). All these findings indicate that the disproportionate number of Vκ and Vλ genes in different species is not the cause of differential Vκ and Vλ usage, but the effect of different rearrangement order and/or developmental dynamics.

Another important factor is the limited number of V_L_ and/or J_L_ gene segments, which approaches zero. If species can rely on one type of IgL, the second may gradually goes unused or be eliminated. This is the case in birds, which have been able to do so probably because they use gene conversion (42–44), but also in bats, which have expanded the Vλ and V_H_ repertoire (60). These are two known species that have eliminated IgLκ (**Table 1**). The opposite might be a case in mice, which use 95% of IgLκ and limited the number of Vλ genes to only two or three (28). Indeed, mice are very specific in the IgLλ locus and use two tandem VλJλCλ cassettes (58) instead of multiple Vλ genes arrayed upstream of JλCλ cassettes as known from other species (21). In any case, ungulates are also interesting as described earlier. In general, these species have sufficient numbers of putative Jκ and Jλ segments, but many of them are mutated and not useful for functional rearrangements. As a result, sheep and cattle have almost no functional Jκ genes. They are either mutated in the W(F)GxG motif and therefore nonfunctional or have noncanonical RSS, making rearrangement inefficient or impossible (59). In comparison, goats have one Jκ segment that is still fully functional, and this may be a reason why they have more IgLλ^+^ B cells than sheep and cattle (59). In this respect, sheep and cattle, followed by goats, might just be other species that follow the bats in the complete loss of IgLκ^+^ B cells. It is surprising that many thriving species are able to keep the number of functional V(D)J segments to a minimum. This is especially true for IgH, which is critical for BCR formation. For example, pigs have all five J_H_ segments “functional” in terms of the WGxG motif and the absence of stop codons (14). However, three of the five have noncanonical RSS (14), and functional experiments have shown that only one of the remaining can be used for functional rearrangement (82). A similar situation appears to apply to goats (59).

### Conclusion

In summary, different species appear to have evolved different strategies for the order of rearrangement of Ig genes and for the selection of a productive Ig repertoire. Possibilities for these strategies have even been indicated in mice themselves, which showed that the order of rearrangement is independent (27, 40, 41) and SLC is unnecessary (47, 48). Apparently, all these species have survived with comparable success. On the other hand, differential regulation of rearrangement order and mechanisms of repertoire selection may have evolutionary and practical consequences. In the IgL-before-IgH group, extensive editing of the IgLλ repertoire occurs very early and before IgH rearrangement. On the other hand, IgLλ repertoire is edited in the IgH-before-IgL group only when IgLκ is unsuccessful. These principles could lead to higher diversification of IgLλ loci in the IgL-before-IgH group, while higher diversification of IgLκ loci in the IgH-before-IgL group. Another consequence of the enormous differences between species is the possibility of choosing the uncomplicated experimental systems for practical purposes. All porcine V_H_ genes share the same leader and framework sequences and only one J_H_ segment is functional. Furthermore, both porcine IgLκ and IgLλ loci contain only two families and two functional J_L_ genes. This allows the recovery of all VDJ_H_ rearrangements using a single non-degenerate primer set or the generation of deficient pigs for B cells by modifying just a single J_H_ segment (82). However, it must always be considered if the regulatory components of Ig rearrangement also need to be copied into the genome. The production of B-cell-deficient pigs may be a simple task, but it may be difficult to generate genetically modified pigs that produce a sufficient amount of human antibodies.

## Author Contributions

All authors listed have made a substantial, direct, and intellectual contribution to the work, and approved it for publication.

## Funding

The research was supported by the Czech Science Foundation grants No. 19-01504S and No. 20-03282S.

## Conflict of Interest

The authors declare that the research was conducted in the absence of any commercial or financial relationships that could be construed as a potential conflict of interest.

## Publisher’s Note

All claims expressed in this article are solely those of the authors and do not necessarily represent those of their affiliated organizations, or those of the publisher, the editors and the reviewers. Any product that may be evaluated in this article, or claim that may be made by its manufacturer, is not guaranteed or endorsed by the publisher.
